# Establish Stronger Correlation between Surrogate Endpoint and Clinical Endpoint Using All Post‐baseline Data

**DOI:** 10.1002/alz.091817

**Published:** 2025-01-09

**Authors:** Yan Li, Guoqiao Wang

**Affiliations:** ^1^ Washington University School of Medicine, St. Louis, MO USA; ^2^ Washington University in St. Louis, School of Medicine, St. Louis, MO USA

## Abstract

**Background:**

A traditional method for validating a surrogate endpoint typically involves assessing the correlation between changes in biomarkers and changes in clinical endpoints using Pearson’s and/or Spearman’s correlation. However, this approach may not provide an accurate representation of the true correlation due to several reasons: (i) it only considers the change from baseline to the last visit and does not use all post‐baseline longitudinal data; (ii) the raw change has large variability; (iii) it does remove the within‐subject variability. The objective of this presentation is to propose two alternative approaches that overcome these limitations and allow for a more accurate estimation of the true correlation using all available longitudinal data.

**Method:**

We propose two models that have the capability to eliminate within‐subject variability and utilize all available post‐baseline longitudinal data for estimating the correlation: (i) bivariate linear mixed effects (bivariate‐LME) model utilizing time as a continuous variable, and (ii) bivariate MMRM with random effects (bivariate‐MMRM‐RE) utilizing time as a categorical variable. Through simulations that mimicking treatments with disease modifying effects, we will compare the estimated correlations using these two models to the Pearson’s and/or Spearman’s correlations.

**Result:**

Our preliminary simulations showed that the Pearson’s and/or Spearman’s correlation tends to significantly underestimate the true correlation. And the magnitude of underestimation increases with shorter trial durations. On the other hand, correlations estimated from both the bivariate‐LME model and bivariate‐MMRM‐RE model demonstrate higher accuracy compared to the true correlation estimation, and they are also less influenced by the trial duration.

**Conclusion:**

The Pearson’s and/or Spearman’s correlations estimated from clinical trial data are usually small (eg, 0.19 between the change in amyloid PET and CDR SB in EMERGE). Our simulations suggest that the Pearson’s or Spearman’s correlation is smaller than the true correlation. Therefore, it is advisable to estimate the true correlation using either the bivariate‐LME model or the bivariate‐MMRM‐RE model. These models provide more accurate estimations of the true correlation compared to the traditional Pearson’s and/or Spearman’s correlation.

